# Understanding the Social Contagion Effect of Safety Violations within a Construction Crew: A Hybrid Approach Using System Dynamics and Agent-Based Modeling

**DOI:** 10.3390/ijerph15122696

**Published:** 2018-11-29

**Authors:** Huakang Liang, Ken-Yu Lin, Shoujian Zhang

**Affiliations:** 1 School of Management, Harbin Institute of Technology, Harbin 150001, China; hkliang5493@hit.edu.cn; 2 Department of Construction Management, University of Washington, Seattle, WA 98195, USA; kenyulin@uw.edu; 3 School of Civil Engineering, Harbin Institute of Technology, Harbin 150090, China

**Keywords:** social contagion effect, routine safety violations, situational safety violations, system dynamics, agent-based simulation

## Abstract

Previous research has recognized the importance of eliminating safety violations in the context of a social group. However, the social contagion effect of safety violations within a construction crew has not been sufficiently understood. To address this deficiency, this research aims to develop a hybrid simulation approach to look into the cognitive, social, and organizational aspects that can determine the social contagion effect of safety violations within a construction crew. The hybrid approach integrates System Dynamics (SD) and Agent-based Modeling (ABM) to better represent the real world. Our findings show that different interventions should be employed for different work environments. Specifically, social interactions play a critical role at the modest hazard levels because workers in this situation may encounter more ambiguity or uncertainty. Interventions related to decreasing the contagion probability and the safety–productivity tradeoff should be given priority. For the low hazard situation, highly intensive management strategies are required before the occurrence of injuries or accidents. In contrast, for the high hazard situation, highly intensive proactive safety strategies should be supplemented by other interventions (e.g., a high safety goal) to further control safety violations. Therefore, this research provides a practical framework to examine how specific accident prevention measures, which interact with workers or environmental characteristics (i.e., the hazard level), can influence the social contagion effect of safety violations.

## 1. Introduction

Despite continuous efforts to promote construction safety over the last decades, construction safety has not improved as much as in other industries, and it still suffers from a high rate of injuries and accidents [1,2,3,4]. Such alarming trends are reported by occupational safety statistics throughout the world [3,5,6,7], and construction safety appears to have reached a plateau [4,6,7,8]. Previous accident investigations have revealed that up to 70% of occupational accidents are attributable to worker safety violations [9]. Safety violations have been widely recognized as the main cause of construction accidents [1,4,5,6,8]. Hence, there is heightened interest in correcting the safety violations of workers to further improve safety in the construction industry.

Based on previous behavioral safety investigations, the violation of safety rules and procedures is pervasive among construction workers [2,10]. For instance, Lipscomb et al. [11] reported that construction workers often did not wear fall protection equipment. Maano et al. [12] also found that most construction workers failed to use personal protective equipment (PPE) while performing tasks on site. Researchers have defined most safety violations as intentional but non-malevolent [13,14]. Here, “intentional” means that violations are distinct from human error and are committed intentionally for various purposes, such as saving time [15]. Human errors are mainly derived from informational problems, where the information is forgotten, incomplete, incorrect, or unknown, while safety violations mainly involve motivational factors and are affected by social norms [16]. The term “non-malevolent” means that violators do not intend to cause accidents or damage to the system [17], which is different from malevolent violations such as sabotage [13]. Construction workers often encounter controversial situations where they balance conflicting objectives like safety and productivity [18,19]. From this standpoint, a coworker’s safety violations can be practical and socially contagious within the construction crew, because such deviations from formal safety procedures seem to be well-intentioned and aimed at getting work done. Therefore, individuals can observe the safety violations of coworkers and learn how to behave similarly [18,20].

In this research, the process by which individuals adopt their coworkers’ attitudes, beliefs, or behaviors is called the “social contagion effect” [21]. Coworkers are critical sources of social influence in the group context [22,23,24,25]. Previous empirical research has revealed that individuals are more likely to break safety rules when they perceive more coworker safety violations [18]. However, there is still lack of research on the dynamic mechanism of the social contagion effect of safety violations, which are complex and might be influenced by cognitive, social, organizational, and environmental aspects. Therefore, this research aims to provide an integrated simulation approach that can analyze how different safety management strategies influence individuals’ decisions, and how the individual observes and learns from coworkers’ safety violations at different sites with different hazard levels. The simulation framework integrates both agent-based simulation (ABM) and system dynamics (SD). The combination of the two frameworks can better define and capture the dynamics and uncertainty of systems on construction sites because of their complementary strengths [26]. Specifically, in this model, the worksite is designed with different levels of hazard and workload: ABM is used to represent the decision rules of individual agents (e.g., workers and management) and the interactions among them; SD is employed to capture the system level dynamics (e.g., the influence of safety goals). The proposed framework has the potential to analyze the dynamic social contagion effect of safety violations and to evaluate various safety management strategies before implementation. 

## 2. Literature Review

This section presents the theoretical and empirical evidence for the social contagion effect of safety violations within construction crews. This section also reviews previous research on construction safety simulation and analyzes the necessity of developing a hybrid simulation framework.

### 2.1. Social Contagion Effect of Safety Violations

Coworkers have been regarded as an important source of social influence in a group context [22,23,24,25]. According to Latane [27], social influence is the function of power, proximity, and number of referents exerting their influence. Coworkers tend to possess more field experience, have closer proximity to fellow workers, and outnumber managers and supervisors [28]. Thus, coworkers have more social influence on individuals [29]. Previous studies have explored various aspects of the social contagion effect that coworkers have on individual workers [30,31,32,33,34,35]. For instance, Robinson and O’Leary-Kelly [33] reported a positive relationship between the levels of antisocial behavior exhibited by individuals and the levels exhibited by their coworkers. Pelps et al. [34] noted the social contagion effect of turnover behavior among workers, where coworker turnover related behaviors could influence an individual’s decision to quit. Brummelhuis et al.’s empirical research on absence behavior revealed that individuals were more likely to call in sick when coworkers were often absent [35]. 

Similarly, in the occupational safety domain, coworkers can also exert critical social influence on individuals’ safety-related perceptions and behaviors [18,20]. For instance, Westaby et al. [36] discovered that risk-taking behavior by coworkers is a significant predictor of young workers’ risk-taking tendency. Stride et al. [37] reported that regardless of organizational prescription, individuals may learn safety norms from the unsafe behaviors of their coworkers. McLain [38] showed that an individual’s beliefs of coworkers’ estimates of risk were positively related to their own perceived safety risk. Therefore, safety violations need to be eliminated by considering the social context of work groups, rather than merely focusing on the traditional and individual-oriented control from management and supervisors (e.g., individual penalties and incentives) [5]. Despite the increasing attention that has been paid to the influence of coworkers on individuals’ safety violations, few studies have explored the underlying mechanism of the social contagion effect, through which safety violation norms are formed within a construction crew [18]. 

On construction sites, a continuous tension tends to exist between the demands of accomplishing production tasks and the requirement to protect workers from unwanted injuries or illnesses [19,39,40]. For instance, Mohamed’s empirical research revealed that work pressure is common in the construction industry, which creates a perceived conflict between safety and productivity [40]. Considering the dynamic complexity in the production process, construction workers often need to balance production and safety objectives discretionarily [5]; they may deviate from some safety rules and procedures to get the job done [19]. Therefore, coworkers can serve as critical role models and sources of social information with regard to safety violations, which may influence an individual’s safety-related decisions [18,20]. Individuals may be more inclined to break safety rules when perceiving higher levels of coworker safety violations [18]. 

Liang et al. [18] developed and tested a social contagion model of safety violation, in which coworkers’ safety violations have a direct effect on individuals’ safety violations through the mechanism of social learning [41]; the indirect effect can be explained with social information processing [42]. Both social learning and social information processing theories identify horizontal dynamics through which individuals are motivated to belong to their social group [35]. Liang et al.’s research provided empirical evidence on the social contagion effect of coworker safety violations within a construction crew [18]. However, previous research has not yet produced sufficient understanding of the interactions between the social contagion effect and the dynamic production process, different environmental conditions (e.g., different hazard levels), and safety management interventions. Therefore, this research will develop a hybrid simulation approach to model the dynamic process of the social contagion effect of safety violations and to explore the effectiveness of various safety management strategies and onsite conditions.

### 2.2. Hybrid Modeling and Simulation Method

A wide range of dynamic simulation models have been developed in previous studies to deal with the complexity of safety-related behaviors on construction sites; among these, system dynamics (SD) and agent-based modeling (ABM) are the most commonly used [3]. Goh et al. [43] demonstrated the influence of production pressure on safety through the SD model. Similarly, Han et al. [44] applied the SD to explore the processes by which production pressure influences onsite safety performance. Shin et al. [1] developed an SD model to capture construction workers’ mental processes to analyze the feedback mechanisms that determine safety attitudes and behaviors. Jiang et al. [2] extended Shin et al. [1] work by taking into account many more underlying factors to create a holistic causation model of construction workers’ unsafe behaviors. In contrast, several studies on safety-related behavior have employed ABM to capture the adaptive decisions and actions of construction workers. Lu et al. [7] observed the relationship between safety investment and safety performance as a complex adaptive problem. They used ABM to investigate how the interactions between the worksite environment, individual workers, and different types of safety investments influenced safety [7]. Choi et al. [5] developed a socio-cognitive model based on ABM that integrated social influence into the cognitive process to explore methods to control construction workers’ unsafe behaviors. Although these simulations have greatly contributed to understanding the underlying mechanism of safety-related behaviors, they used either SD or ABM alone. Hybrid studies that can combine the strengths of SD and ABM in handling the dynamic complexity of safety problems on construction sites has not yet been fully explored.

The SD is a top-down approach for representing a homogenous system that is described by the feedback structure from an aggregate level [45]. By contrast, as a bottom-up approach, the ABM can capture the heterogeneity of agents at the individual level but has limitations in modeling the macro system [26,46]. As evidenced by previous studies on the consumer choice behaviors of sustainable products [46,47], the diffusion process of innovative technologies [48], and the impact of alternative economic policies on water use and pricing [49], the different mechanisms of SD and ABM mean that they can have complementary roles and, therefore, achieve a better understanding of complex systems [26]. Nasirzadeh et al. [50] have developed an SD-ABM simulation model to investigate the social influence on safety violations. However, their model mainly focused on the safety-related interactions between different onsite contractors without paying sufficient attention to the social contagion effect of safety violations within a construction crew. Moreover, above study did not take into account the critical roles of the management and the environmental factor (i.e., the hazard level) [3,50]. Therefore, it is necessary to develop a hybrid SD-ABM simulation model that represents the system and individual level dynamics to explore the social contagion effect of safety violations in detail and to quantify the effectiveness of various interventions.

## 3. Development of the Hybrid SD-ABM Simulation Approach

This research proposes a hybrid SD-ABM simulation approach to understand the social contagion effect of safety violations. The SD method captures the system level dynamics, which are derived from the cause–effect relationships between the macro system factors. For instance, increasing accidents can lead to a higher accident-control pressure and a much stricter management in terms of safety violations. On the other hand, a low production rate can cause an increased production pressure, making workers more prone to commit safety violations. The ABM method describes the heterogeneous individual worker interactions with other components (e.g., coworkers and management) and the consequences of behaviors (e.g., whether a violation can cause an accident or not) in the construction project. 

**Figure 1:**
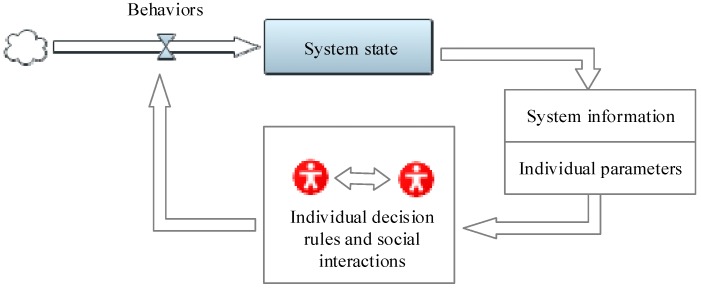
The conceptual framework of the hybrid simulation approach.

The conceptual framework of the hybrid simulation approach was established on the basis of Sterman’s general priority for combining agents into the SD method [51]. As shown in Figure 1, this framework considers two different parts: (1) the system level dynamics and (2) the safety-related decision rules and the social interactions of the agents. The system level dynamics generate the information that can influence the safety-related decisions of agents (e.g., the social support environment decreases workers’ safety violation tendency). The behavior resulting from individual decision processes can consequently change the system state (e.g., the amounts of accidents caused by safety violations) and alter the information from the system (e.g., accident control pressure), which will further affect individual agents. The cycle of information exchange between the system and individual parts operates continuously, which determines the dynamic social contagion effect of safety violations. The hybrid SD-ABM model will be developed using the Anylogic software. Anylogic is a reliable Java-based simulation tool that has functional advantages in combining SD and ABM [52,53]. 

### 3.1. Defining the Virtual Construction Environment

**Figure 2:**
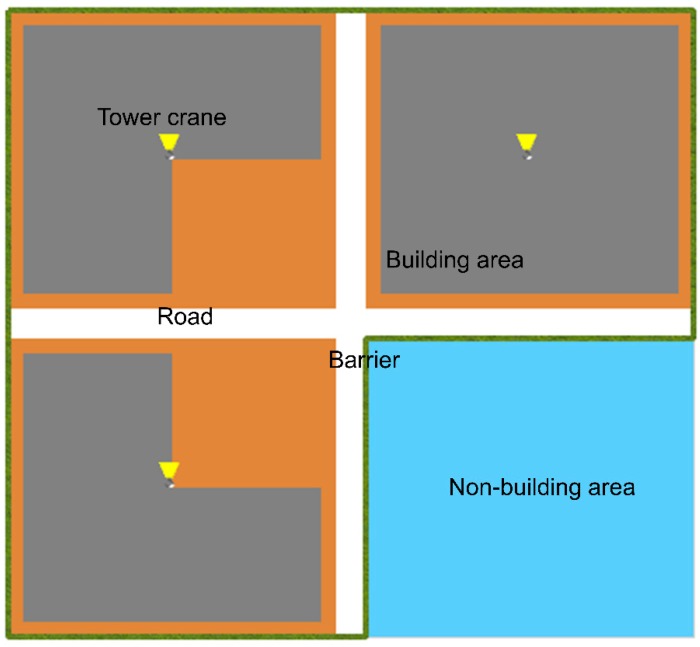
Virtual construction site.

Previous construction simulation studies have tended to use cells or grids to represent the workplace, because construction activities can always be divided into basic tasks dispersed around the site with varying degrees of workloads and hazards [7,54,55]. Therefore, a typical cell-based virtual construction site adapted from Lu et al. [7] is employed to represent main onsite characteristics. As shown in Figure 2, this virtual construction is constituted by 92 × 84 cells of 1 m^2^ each; while only the gray areas represent buildings under construction (totaling 3600 cells of 1 m^2^ each). Each cell demands a certain workload varying from 0 (no workload) to 20 (heavy workload) and has a hazard level from 0 (a very safe place where no accidents will happen) to 200 (a very dangerous place where associated safety protection activities should be performed to avoid unwanted accidents and injuries). There are two main temporary roads for transporting construction materials. Three tower cranes are key sources of dynamic hazards on the construction site; therefore, a dynamic danger zone with cells under the crane jib is generated, which have hazard levels varying between 160 and 200. 

### 3.2. Defining the Decision Rules and Social Interactions of Agents

The decision rules and social interactions between heterogeneous agents are modeled using ABM. Specifically, the components of ABM in this research include the decision rules for safety violations, the interactions between management and workers, and the social contagion effect of safety violations.

#### 3.2.1. Decision Rules for Safety Violations

**Figure 3:**
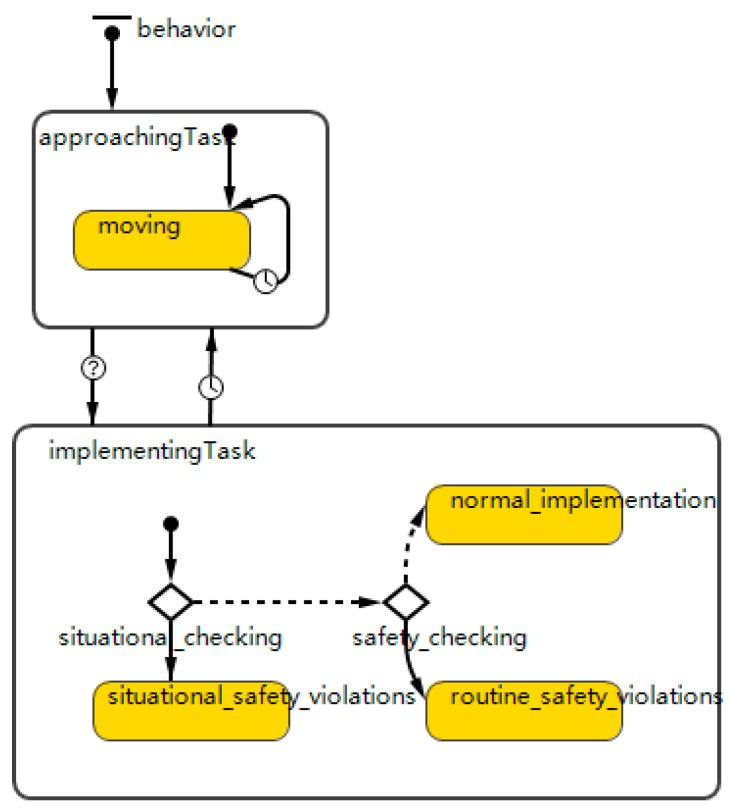
State chart for representing the decision rules of safety violations.

This research assumes that worker agents have two main states, namely, approachingTask and implementingTask (shown in Figure 3). ApproachingTask identifies that if the cells occupied by workers have 0 workload, the worker agents will move to another cell nearby. Once the workers reach a cell with a workload, they will begin to operate the workload and the worker state will transfer from approachingTask to implementingTask (shown in Figure 3). Considering that accidents and injuries often occur during task implementation rather than the approaching process [56], this research mainly focuses on safety violations during the task implementation. 

Safety violation herein refers to a specific way of task implementation, where individuals work around the blocks or barriers implemented in tasks to get jobs done faster [57]. Some blocks are intentionally included as important controls (e.g., the safety rules and procedures) to improve safety. In contrast, other blocks or barriers (e.g., lack of proper safety resources and adverse work environment) are unintentional; these are not anticipated in the design of the work procedure. This research regards such unintentional blocks as “situational constraints” and categorizes safety violations into routine and situational violations based on whether situational constraints are the main cause for violations [18]. Routine violations occur when workers work around some safety procedures in order to realize organizational benefits (e.g., getting the job done in a timely manner) or personal gain (e.g., saving time or effort). In contrast, situational violations tend to be driven by situational constraints in tasks, which make it difficult or impossible to follow the rules [15]. 

Worker agents will first check the working environments to verify the existence of situational constraints via situational checking (shown in Figure 3). This research assumes that if there is the presence of situational constraints, worker agents will commit situational safety violations. If there is no situational constraint, they will further compare the hazard level in the current cell with their acceptable hazard level via safety checking (shown in Figure 3). According to Wilde’s theory of risk homeostasis [58], perceived hazard levels and acceptable hazard levels are the two main dimensions that determine risk-taking behaviors. Specifically, construction workers will commit routine safety violations if the perceived hazard level is lower than the acceptable value; otherwise, they will be compliant with the safety procedure and proceed with normal task implementation. The acceptable hazard level of each worker is controlled by a variable acceptable hazard. After going through the above decision-making process, workers will select a specific way to implement the task. The implementation time is determined by the workload level in the current cell and the workers’ production speed controlled by the variable production speed. Therefore, the worker state will change from implementing task to approaching task after the time delay needed to complete the current task.

There are three possible outcomes when safety violations are committed. In the first outcome, there is no accident; instead there is an increased production speed since the worker has made a tradeoff between safety and production, sacrificing safety for productivity. The increased rate of production is determined by the variable productionIncr (i.e., production increase). The second outcome is a near miss incident, which involves a dangerous situation that has the potential to generate accidents, but that does not cause injuries or material damage [59]. In the third outcome, the worker agents will suffer from injuries or accidents and will consequently stop their work for three days to represent the production loss caused by accidents [7]. A previous accident experience can also negatively influence workers’ attitude toward safety, namely, their acceptable hazard level [37]. This research assumes that the acceptable hazard level will decrease by 5 or 50, if the worker agent experiences a near-miss incident or accident, respectively. 

#### 3.2.2. Interactions between Management and Workers

The interactions between frontline management and workers are critical for the workers’ perception of the formal safety rules established at the organizational level, which further influences their decisions to balance safety and productivity [60]. Safety feedback and safety improvement are two main supportive behaviors from management to prevent worker safety violations onsite [61]. In this research, safety feedback refers to the warnings from the management when they find that workers are not following safety rules. By contrast, a safety improvement is the corrective action from management where they find and remove situational constraints that workers are exposed to (e.g., make PPE much more available for workers). If the situational constraint is addressed by the management, the workers will not commit situational safety violations (shown in Figure 3). This research assumes that management initiates safety feedback and safety improvement only when the hazard level that workers are exposed to is higher than their tolerance. This means that they will not make any feedback toward the safety violations or make any corrective actions toward the situational constraints if they think the hazard level is very low. In addition, this research assumes that management can only modify safety violations and situational constraints within a limited distance and also only within a specific probability lower than 1.0, reflecting different management capacities or the limited management energies allocated to safety. These factors are represented by the variables distance, safety feedback rate, and safety improvement rate.

#### 3.2.3. Social Contagion Effect of Safety Violations

Regarding the interactions between construction workers, this research mainly focuses on the social contagion effect of safety violations. The social contagion effect refers to the process by which individuals adopt coworkers’ safety-related attitudes and behaviors [21], which is critical for the formation of safety-related norms within a construction crew. Previous empirical studies have revealed that coworkers’ safety violations have a social contagion effect on individuals [18]. When more coworker safety violations are perceived, individuals will have a higher hazard acceptance level because they tend to adapt themselves to the social norm within construction crews [18]. Specifically, individuals can perceive a tolerable hazard level within their crews based on the interpretation of their coworkers’ safety violations and their memories of their coworkers’ past behaviors. Previous studies have tended to use memory capacity in the process of perceiving social norms [5,62]. In this research, perceived tolerable hazard levels within a crew is the weighted sum of previous hazard tolerance levels and the current average hazard coworkers are exposed to when they implement tasks (shown in Equations (1) and (2)):

(1) $$TC_{i}^{\left(t\right)}=\left(1-\frac{1}{m}\right)TC_{i}^{\left(t-1\right)}+\frac{1}{m}\left(\frac{1}{c_{i}^{\left(t\right)}}\overset{c_{i}^{\left(t\right)}}{\underset{c_{i}=1}{\sum }}HA_{c_{i}}^{\left(t\right)}\right))$$

(2) $$HA_{c_{i}}^{\left(t\right)}=\frac{1}{t_{c_{i}}^{\left(t\right)}}\overset{v_{c_{i}}^{\left(t\right)}}{\underset{v_{c_{i}}=1}{\sum }}HA_{v_{c_{i}}}^{\left(t\right)}$$

where $TC_{i}^{\left(t\right)}$ denotes the perceived hazard tolerance level within a crew at time t; $TC_{i}^{\left(t-1\right)}$ denotes the perceived hazard tolerance level in time t − 1; m represents individual’s memory capacity regarding coworker safety violations; $HA_{c_{i}}^{\left(t\right)}$ represents the average hazard level the $c_{i}^{\left(th\right)}$ coworker is exposed to during task implementation at time t; $HA_{v_{c_{i}}}^{\left(t\right)}$ is the hazard level the $c_{i}^{\left(th\right)}$ coworker is exposed to during the $v_{c_{i}}^{\left(th\right)}$ violation; $c_{i}^{\left(t\right)}$ is the coworker number of individual i at time t; $v_{c_{i}}^{\left(t\right)}$ is the total number of safety violations of the $c_{i}^{\left(th\right)}$ coworker at time t; and $t_{c_{i}}^{\left(t\right)}$ represents the total number of tasks (i.e., the number of cells that this worker passes through) completed by the $c_{i}^{\left(th\right)}$ coworker at time t. 

However, the influence from coworkers can be affected by formal safety rules [5,62], namely, the hazard tolerance level of management, which is represented by $TM^{\left(t\right)}$. According to Ahn et al. [62], individuals form their own internal rules for behaviors by combining formal rules from management and the informal social norms from coworkers. This research defines $TR_{i}^{\left(t\right)}$ as the final perceived hazard tolerance level, determined by a weighted sum of the hazard tolerance level in the crew $TC_{i}^{\left(t\right)}$ and the hazard tolerance level of management $TM^{\left(t\right)}$ (shown in Equation (3)). $w_{i}^{\left(t\right)}$ represents the attitudinal ambivalence toward safety compliance, which refers to the extent to which an individual simultaneously holds positive and negative attitudes toward safety compliance [63]. When workers have higher attitudinal ambivalence, they are more likely to be influenced by coworkers’ safety violations, because they have not yet established firm beliefs regarding safety behaviors [18]:

(3) $$TR_{i}^{\left(t\right)}=w_{i}^{\left(t\right)}TC_{i}^{\left(t\right)}+\left(1-w_{i}^{\left(t\right)}\right)TM^{\left(t\right)}$$

Then, individuals will adapt their acceptable hazard level to perceived hazard tolerance as established by the coworkers’ safety violations and formal safety rules; the adaptation process is shown in Equation (4). Here, the contagion probability is a parameter representing the extent to which individuals will be influenced by coworker safety violations and formal safety rules. The contagion probability ranges from 0 to 1, where 0 denotes individuals that do not change their behaviors even under external influences, while 1 describes individuals that are completely determined by external influences. Therefore, this variable helps capture situations where individuals might have different responses to the same external influences [52]:

(4) $$AR_{i}^{\left(t\right)}=\left(1-contagion\;probability\right)*\;AR_{i}^{\left(t-1\right)}+contagion\;probability*TR_{i}^{\left(t\right)}$$

### 3.3. Defining the System Level Dynamics

**Figure 4:**
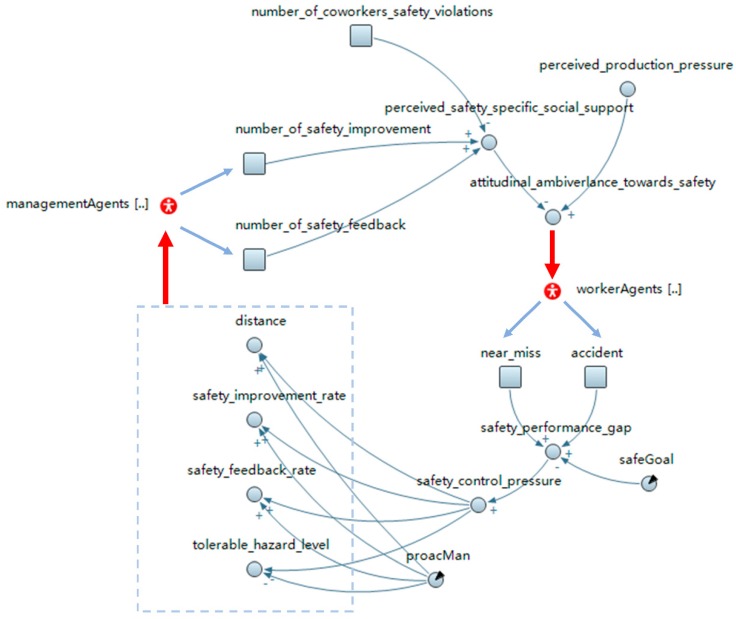
Feedback structure of the system level dynamics.

In this research, the system level dynamics are modeled via SD, the structure of which is shown in Figure 4. The specific description and calculation process are presented in Table 1. The stocks (i.e., the square blocks) represent a change of the worker state (e.g., near-miss and accident) and the number of certain types of interactions between management and workers (e.g., safety improvement number and safety feedback number). Unlike the traditional single SD model, the rate of change in a stock is determined by agent behaviors (shown in Figure 1, where behaviors influence the system state). Nevertheless, the stocks play the same role as in the traditional SD model because they describe variables that accumulate or deplete over time [26]. Some intermediate variables are introduced and determined by other variables in the calculation process. For example, this research regards the ratio of the total number of safety improvements and the safety feedback received by coworkers within the crew from the management to the total number of coworkers’ safety violations as a proxy of the perceived safety specific social support. This means that when individuals observe more interactions between management and their coworkers, and there are less coworker safety violations, they will perceive higher safety specific social support. This assumption is based on previous empirical studies that showed that individually perceived safety-specific social support is inversely related to coworker safety violations [18]. Based on the path coefficients in the social contagion model established by a previous empirical study [18], the ambivalence toward safety compliance is determined by the weighted sum of the perceived safety-specific social support and the perceived production pressure. Some variables are predefined inputs such as safeGoal and proacMan, which represent the safety goal and the intensity of proactive management strategies, respectively. The outputs are generated for different agents (shown in Figure 4). For instance, attitudinal ambivalence toward safety compliance is an output for worker agents, whereas the safety improvement rate, safety feedback rate, and tolerable hazard level are outputs for the management agents. These outputs will become the inputs of the agents, consequently alter their behaviors, and further influence the state of the system (shown with the red lines in Figure 4).

## 4. Initialization and Validation of Baseline Model

Before performing the simulations, the simulation model is verified and validated. First, the baseline model is developed by initializing the parameters regarding the system level dynamics, the safety-related decision rules, and the social interactions. Next, a combination of verification and validation techniques are applied to ensure the robustness and technical validity of the baseline model.

### 4.1. Initialization

There are 100 workers on the simulated construction site (shown in Figure 2), consisting of 5 work crews with 20 workers in each work crew. According to the investigation by Lu et al. [7], a safety supervisor usually inspects 20 workers on a construction site. Therefore, the number of managers is initially five. Before each run of the simulation, the parameters acceptableHazard (i.e., acceptable hazard level) and contagionPro (i.e., contagion probability), which describe workers’ acceptable hazard level and how they are affected by the social interactions, respectively, are assigned different values to represent the workers’ heterogeneous attributes. These parameters are assumed to follow a uniform distribution, because the uniform distribution tends to be the most appropriate when the distribution is unknown [59]. The range of acceptableHazard is from 20 to 180 to exclude extreme cases where workers are absolutely risk-averse or risk-seeking. Similarly, the range of contagionPro is set from 0.1 to 0.9.

Breaking safety rules means that workers need to make a tradeoff between safety and productivity, in other words, sacrificing safety to get the job done in a timely manner [18]. In this research, the variable productionIncr (i.e., a production increase) is used to describe the increased production when there is a safety–productivity tradeoff. This tradeoff reflects the organizational characteristics where there is an unsuitability between safety and productivity onsite. The productionIncr herein is conservatively assumed to be 0.2, which means that workers’ production speeds may be temporarily increased by 20% when safety rules are violated. In addition, we assume that, under normal task implementation (shown in Figure 3), one construction worker has a daily productivity of 20. In this research, construction workers develop their perception of coworkers’ attitudes toward safety violations (i.e., their tolerable hazard level) through observations of current coworker safety violations and through the memory of their past behavior [5]. Workers are assumed to remember 28 days of coworker safety-related behaviors on sites. In addition, as mentioned earlier, situational constraints are critical causes for situational safety violations [18]. The probability of workers being exposed to situational constraints is simply controlled by the task complexity, namely, the workload in each cell. Lastly, workers within the same crew tend to interact much more frequently with each other than with members in different crews. Thus, this research assumes that workers can observe other workers within their own crews (i.e., clique), while social interaction across crews is limited (i.e., a sparse network) [5,62]. 

In this research, management behaviors are controlled by four parameters: safety_feedback_rate, safety_improvement_rate, distance, and tolerable_hazard_level (shown in Figure 4). The values of these variables are determined by both the proacMan and safeGoal. The proacMan variable describes the proactive safety management strategies that are implemented before the occurrence of any negative safety outcomes (i.e., near-misses and accidents) [64], whereas safeGoal can be regarded as the reactive response to poor safety performance which can influence the above four parameters through safety control pressure (shown in Figure 4). The values of proacMan and safeGoal represent the difference in the project safety culture. For the baseline model, the proacMan value is set as 0.5, and, accordingly, the initial values of the safety_feedback_rate, safety_improvement_rate, distance, and tolerable_hazard_level are 0.5, 0.5, 5, and 50, respectively. This means that management makes safety improvement for situational constraints or gives safety feedback for safety violations by a probability of 0.5. In addition, management has a tolerable hazard level of 50 and an inspection distance of 5 m. By contrast, the safeGoal is initialized as 1.25 in the baseline model. Lastly, the distribution of the onsite hazard level for the baseline model is set at a modest level by a triangular distribution of (0,100,200). 

### 4.2. Verification and Validation

Verification is the process of determining whether the implementation of the simulation model is correct. Meanwhile, the validation is the process of determining whether the model has made a reasonably true representation of the real world for the purpose of addressing the research questions [65,66]. A combination of commonly used verification and validation techniques in recent construction simulation literature are adapted in this research [5,52,53]. 

#### 4.2.1. Model Verification

The model verification was implemented by following the guidelines of Raoufi et al. [53] and Azar et al. [52], because they also addressed a construction simulation based on the Anylogic software. First, all mathematical equations (e.g., Equations (1)–(4)) were computed manually, whose results were compared with the values calculated by the model [65]. Second, a code walkthrough and a debugging walkthrough were performed to identify any possible programming errors [67]. Third, it was ensured that the model components work as expected by closely observing and tracing the changes in variables and the interactions of individual agents (e.g., worker agents) throughout the duration of the simulation [67].

#### 4.2.2. Model Validation

There are numerous methods with different levels of rigor that can be used for the validation of simulation models. For instance, Zeigler et al. [67] distinguished three different types of validity: replicative validity (i.e., “the model matches data already acquired from the real world”), structural validity (i.e., “the model truly reflects the way in which the real world operates”), and predictive validity (i.e., “the model matches data before being acquired from the real world”). Appropriate validation methods can be determined according to the available data and the model’s intended purpose [67]. The objective of this research was to explore the social contagion effect of safety violations by considering the behavioral dynamics from both the individual level and the system level, rather than to provide an accurate pinpoint prediction of safety violations. Therefore, this research mainly focused on the replicative validity and the structural validity [5]. First, both the quantitative and qualitative agreements between the simulation model and the empirical macrostructures of the subject were examined to ensure the replicative validity [5]. Specifically, the model results were compared with previous empirical studies to examine the qualitative agreements of the model [5,68]. Then, national non-fatal injuries data were used to validate the quantitative agreement of the model [5]. Finally, some commonly used techniques (e.g., sensitivity analysis) were performed to enhance the structural validity [68]. 

**Figure 5:**
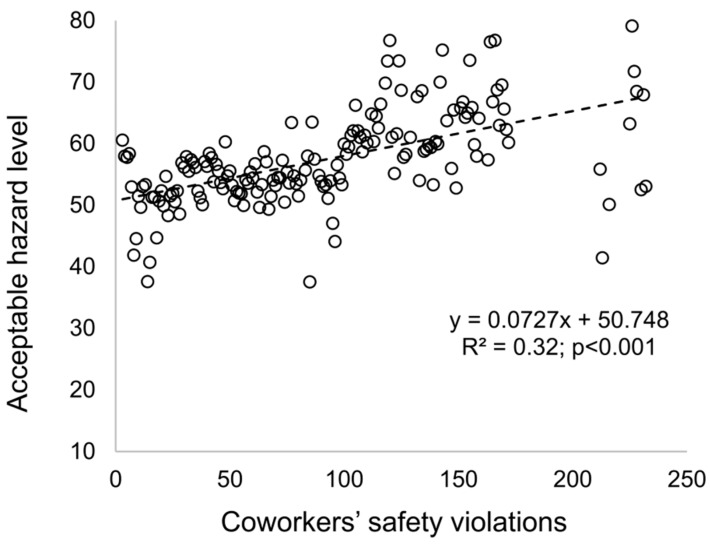
Relationship between the number of coworker safety violations and the individual acceptable hazard level.

The qualitative agreement of the replicative validity was first examined. Figure 5 shows that coworkers’ safety violations have a positive effect on individuals’ acceptableHazard level in the baseline model (R^2^ = 0.32; *p* < 0.001). This result can be supported by social influence studies showing that individuals adapt to social norms by observing how their coworkers behave in groups [34,35]. These include safety-related behaviors since Liang et al.’s previous empirical research revealed that, within a construction crew, individuals who perceive more coworkers’ safety violations are more likely to break safety rules [18]. As mentioned earlier, construction workers often encounter a conflict between safety and productivity onsite [19]. As such, when coworkers break safety rules intentionally to get the job done, individuals may observe these behaviors and learn how to operate in similar situations [18]. 

**Figure 6:**
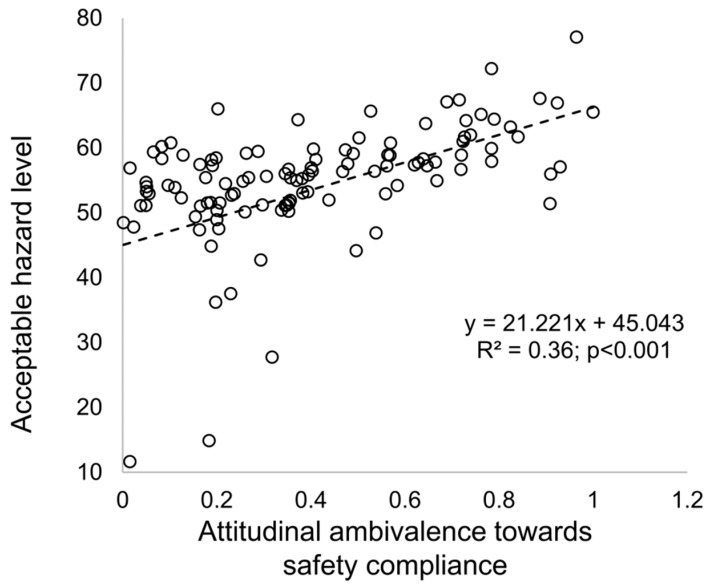
Relationship between attitudinal ambivalence toward safety compliance and the individual acceptable hazard level.

**Figure 7:**
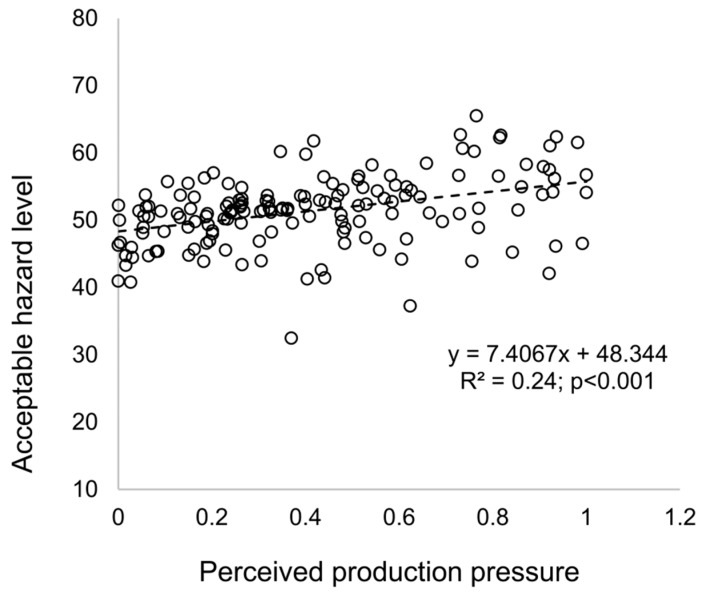
Relationship between the perceived production pressure and the individual acceptable hazard level.

The simulation results can also reproduce results from previous empirical studies on the effect of attitudinal ambivalence toward safety compliance and safety violations [18,69,70]. As shown in Figure 6, attitudinal ambivalence toward safety compliance has a positive relation with the acceptable hazard level (R^2^ = 0.36; *p* < 0.001). Safety violations are often the result of attitudinal ambivalence due to the contradiction between perceived cost and benefit. In other words, despite the potential benefits, workers are often reluctant to follow onsite safety rules due to the immediate costs, such as slower pace, extra efforts, or personal discomfort [71]. Workers with high attitudinal ambivalence are receptive to opposing the views of organizational safety procedures and are more likely to violate safety rules [18]. In addition, the baseline model also strongly confirms previous arguments that the perceived production pressure is a critical cause of safety violations on construction sites [6,43,72,73,74]. As illustrated in Figure 7, the perceived production pressure is positively related to the acceptable hazard level (R^2^ = 0.26; *p* < 0.001). Production pressure is common onsite because of the high interdependency between construction processes: a delay in one area can cause costly delays in others [18]. Workers who perceive high production pressure will focus their attention more on how to complete tasks quickly and cut corners more readily to avoid poor production performance [6,18]. 

It is noted that the values of R^2^ obtained from the above three linear regressions are not very high. This suggests that more variables should have been included in these regressions. Nevertheless, the significant p values in these regressions still indicate a real relationship between these three variables (i.e., number of coworkers’ safety violations, attitudinal ambivalence toward safety compliance, and perceived production pressure) and individuals’ acceptable hazard level.

To examine the quantitative agreement between the simulation results and empirical data, the baseline model was executed 50 times, and the average values of several important indicators were calculated (shown in Table 2). These simulation results were compared with empirical data from previous studies. First, the average ratio of safety violations (i.e., the number of safety violations per worker per day) was 0.32 (standard error σ=0.090), which is consistent with the findings of Sa et al. [75] and Fang and Wu [76]. Both studies reported that one-third of workers did not follow safety rules on construction sites [75,76]. Second, the proportion of situational safety violations was 0.11 (standard error σ=0.028); this can be supported by the investigation of Man et al. [15], which found that 13% of safety violations are caused by situational constraints on site. Third, the ratio between near-misses and accidents (equal to 1:8.18) approximately followed the Heinrich triangle, which proposed that the ratio between accidents (including major and minor accidents) and near-misses is approximately 1:10 [5,77]. Finally, the average rate of accidents (i.e., the number of accidents per 100 full-time workers) was 3.16 (standard error σ=2.136), which is very close to the national incidence rate of occupational injuries and illnesses in the construction reported by the U.S. Bureau of Labor Statistics in 2016 (equal to 3.2) [78]. Therefore, the quantitative agreement between the simulation results and empirical data can be validated.

In addition to the replicative validity, the structural validity of the baseline model was enhanced by the following four steps. First, well-established theories in social sciences (e.g., social learning [41] and social information processing [42]) and validated behavioral dynamic models (e.g., [5,52,62]) were adapted to describe the interaction rules among agents (i.e., workers and management). Second, the casual loops in the system level (e.g., the determination of attitudinal ambivalence) were developed mainly according to Liang et al. [18] previous empirical study. Third, the model parameters (e.g., contagionPro and acceptableHazard) were initialized primarily according to previous empirical studies or common principles used in construction safety simulation (e.g., [5,7]). Finally, the sensitivity analyses were performed on the main parameters (i.e., safeGoal, proacMan, median contagionPro, and productionIncr) to ensure that the model reacts to individual parameter changes in a realistic manner. The values of these parameters were increased by 50% over their base value individually. Then, the simulation model was run 50 times for each variation. The percentage changes of the average values of the main model outputs are presented in Table 3. All above parameters can influence the simulation results in the expected directions. First, increases in each parameter can all lead to worse safety-related outputs (i.e., ratio of safety violations, rate of accidents, and rate of near-misses). Among all the parameters, proacMan is the most influential factor for these safety-related outputs. It was also observed that the rate of productivity only has a slight increase, because the productivity cannot be increased significantly by violating more safety rules. Based on the above techniques, the structural validity of the baseline model can be better ensured. 

## 5. Factorial Experimental Design and Simulation Results

Factorial experimental design (FED) is commonly used by social scientists because it has the advantage of testing the impact of a single variable as well as the potential interactions between two or more variables simultaneously [79]. FED is also important for addressing safety issues because the possible combination of interventions to improve safety performance is often not clearly stipulated [3]. Therefore, in this research, FED will be applied to determine influential safety interventions and their potential interactions.

### 5.1. Factorial Experimental Design

In this research, 2^k^ FED was performed to evaluate the main effects of four controllable factors (i.e., safeGoal, proacMan, median contagionPro, and productionIncr) and their interactive effects on construction workers’ safety behaviors. There are two credible levels (positive and negative) for each of the above four key factors (shown in Table 4). Considering that there are four factors and that each can be varied based on two levels, a total of 16 possible factor combinations can be obtained (shown in Table 5). For each of the 16 intervention configurations, the simulation was run 50 times and the average values of the main response variables (i.e., rate of accidents, ratio of routine safety violations, ratio of situational safety violations, and rate of productivity) were calculated. In addition, different site conditions (e.g., the hazard level) can be a source of interaction with the abovementioned 16 possible intervention combinations and can further influence the social contagion effect of safety violations. Therefore, the factorial experimental design will be implemented with three different hazard levels (i.e., low, modest, and high) to provide more insight on effective safety management strategies for different site conditions. As mentioned earlier in Section 4.1, the hazard was assigned based on a triangular distribution where the low hazard level was set at (0,50,200), the modest hazard level was set at (0,100,200), and the high hazard level was set at (0,150,200). Lastly, the experimental design and analysis of results were carried out using the Minitab 18.0 software package. 

### 5.2. Simulation Results

**Figure 8:**
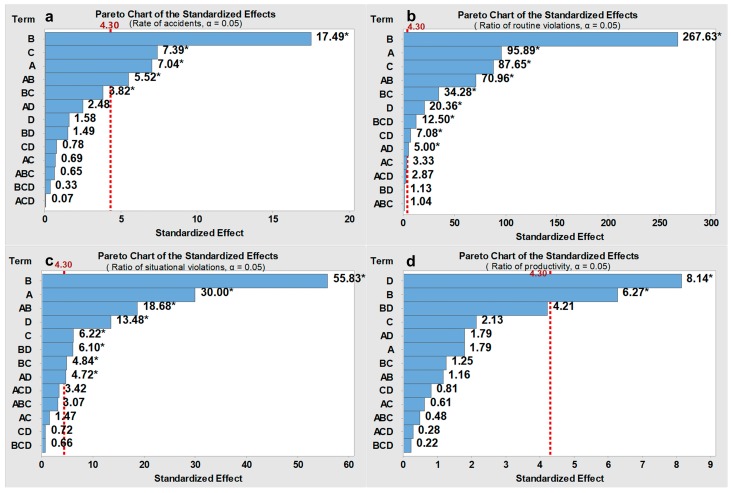
Pareto charts of the standardized effects for the “modest” onsite conditions. Note: (**a**) = safeGoal; (**b**) = proacMan; (**c**) = median contagionPro; (**d**) = productionIncr; * = *p* < 0.05.

**Figure 9:**
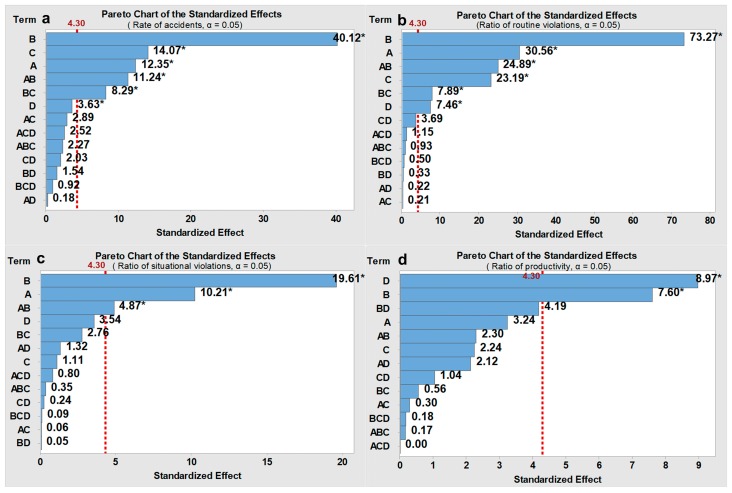
Pareto charts of the standardized effects for the “high” hazard onsite conditions. Note: (**a**) = safeGoal; (**b**) = proacMan; (**c**) = median contagionPro; (**d**) = productionIncr; * = *p* < 0.05.

**Figure 10:**
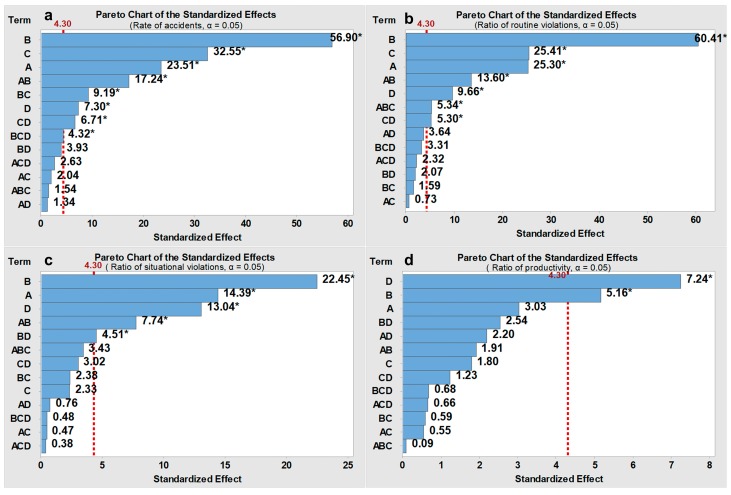
Pareto charts of the standardized effects for the “low” hazard onsite conditions. Note: (**a**) = safeGoal; (**b**) = proacMan; (**c**) = median contagionPro; (**d**) = productionIncr; * = *p* < 0.05.

The FED was performed for three different onsite conditions (i.e., the high, modest, and low hazard levels), and the average values of the response variables (i.e., rate of accidents, ratio of routine violations, ratio of situational violations, and rate of productivity) are captured in Table 6, Table 7 and Table 8, respectively, for each hazard level. The main effects of the four input factors and their interactive effects are evaluated using Standardized Pareto charts (shown in Figure 8, Figure 9 and Figure 10). Pareto charts display a frequency histogram where the length of each bar on the chart is proportional to the absolute value of its associated standardized effect. The minimum statistically significant effect magnitude for a 95% confidence level (*p* ≤ 0.05) is represented by the vertical line in the charts (=4.30). 

The rate of accidents and the ratio of routine violations differed significantly for different intervention combinations, while the ratio of situational violations and the rate of productivity only had slight changes (shown in Table 6, Table 7 and Table 8). Situational violations are triggered by situational constraints, which is different from the cognitive process of routine violations [18]. Although situational constraints can be eliminated by management through safety improvements as mentioned in Section 3, the occurrence of situational constraints tends to be random and transient; therefore, the effect from management is limited. The moderate fluctuations of the rate of productivity are mainly because safety violations cannot improve the productivity dramatically. In the FED, the degree to which one violation can improve productivity (i.e., the productionIncr) varied only from 0.08 to 0.32. The best outcomes regarding the rate of accidents, ratio of routine violations, and ratio of situational violations can be obtained when the safeGoal, proacMan, median contagionPro, and productionIncr are all set to low levels (i.e., design point 1). Here, a lower safeGoal indicates a stricter safety goal, a lower proacMan indicates highly intensive safety management strategies before the occurrence of accidents, a lower median contagionPro indicates a lower probability that individuals will be influenced by coworkers’ safety violations, and a lower productionIncr indicates a lower tradeoff between productivity and safety. By contrast, the worst outcomes can be observed when the safeGoal, proacMan, median contagionPro, and productionIncr are all high (i.e., design point 16). In addition, construction workers may break more routine safety violations as the hazard level decreases [1,15]. 

The Standardized Pareto charts in Figure 8, Figure 9 and Figure 10 are used to illustrate the main effect of each factor and the interactive effects between factors. The results show that the factor proacMan has a dominant-positive effect on three safety-related indicators (i.e., rate of accidents, ratio of routine violations, and ratio of situational violations) for the three onsite conditions. This can be indicated by the largest standardized effects of proacMan (denoted by B) on the associated response variables in Figure 8a–c, Figure 9a–c and Figure 10a–c. By contrast, productionIncr can exert more positive effects on the rate of productivity than other factors, indicated by D in Figure 8d, Figure 9d and Figure 10d. Some interactive effects with statistical significance are found. For instance, the combination of safeGoal and proacMan (denoted by A × B) has a positive effect on three safety-related indicators (shown in Figure 8a–c, Figure 9a–c and Figure 10a–c), which means that a high safeGoal with a high proacMan would lead to worse safety performance. The main effect of the median contagionPro on the ratio of routine safety violations is statistically significant across three hazard levels, and is stronger than those of the productionIncr, which can be seen in Figure 8b, Figure 9b and Figure 10b. However, the median contagionPro have a lower effect on situational safety violations than the productionIncr, which is indicated in Figure 8c, Figure 9c and Figure 10c.

For the ratio of routine violations, proacMan, safeGoal, median contagionPro, and productionIncr have higher effects at the modest hazard level. For instance, the median contagionPro can have a stronger effect on routine safety violations when the hazard level is modest (standardized effect = 87.65, *p* < 0.05, see Figure 8b) than those when the hazard level is high (23.19, *p* < 0.05, Figure 9b) or the hazard level is low (25.30, *p* < 0.05, Figure 10b). This may be because the hazard in the work environment plays a critical role for the onsite conditions characterized by high or low hazard levels (e.g., workers are more likely to not emulate coworkers’ safety violations when the environment is very dangerous, while workers will break safety rules regardless of coworkers’ behaviors in a safe environment). 

The results also indicate the difference in the effects of above four factors on situational safety violations for different hazard levels. For instance, the effects of median contagionPro in the case of the modest hazard level (6.22, *p* < 0.05, Figure 8c) are higher than those for high hazard levels (1.11, not significant, Figure 9c) and low hazard levels (2.33, not significant, Figure 10c). 

## 6. Discussion

This research developed a hybrid SD-ABM simulation approach to understand the social contagion effect of safety violations within construction crews. Well-established theories (e.g., social learning [41] and social information processing [42]), validated models (e.g., [5,58,62,80]), and Liang et al. [18] previous empirical study were used as a reference. The model was operated using the Anylogic software, and associated verification methods were implemented. The model validity was also verified by ensuring that there was an agreement between the baseline model and previous empirical studies both qualitatively and quantitatively and by ensuring the structural validity based on a sensitivity analysis [5]. Lastly, the FED method was employed to assess the main effects of four management strategies (i.e., proacMan, safeGoal, median contagionPro, and productionIncr) and their interactive effects for three different hazard situations (i.e., low-, modest-, and high-levels of hazard). 

### 6.1. Effects of Proactive and Reactive Management Strategies

Previous studies have modeled organizational reactive responses to accidents by setting safety goals (i.e., safeGoal) [2,3]. When setting a higher safety goal, organizations will have a higher accident control pressure for responding poor safety performance (shown in Figure 3). Despite these reactive actions being able to promote safety management systems, proactive management strategies (i.e., proacMan), which are implemented prior to accidents or injuries, should be more important. Organizations that have proactive management strategies are more likely to regard workers’ safety as a core value and maintain a much lower tolerance toward onsite hazards. Such organizations will change safety systems by taking preventive actions, such as decreasing the tolerable hazard level, enlarging the scope of inspections, and increasing the feedback rate. Although setting a high safety goal can lead to much stricter management actions, it is mainly driven by circumstance (e.g., poor safety performance) and cannot ensure the continuity of safety efforts as a proactive strategy. The findings from the sensitivity analysis and the FED indicated that proactive management strategies have a much stronger effect on safety performance (i.e., rate of accidents, ratio of routine safety violations, and ratio of situational violations) compared to setting safety goals. This is consistent with Jiang et al. [2] simulation regarding construction safety, which also reported that preventive measures are much more effective than reactive measures for controlling safety violations [2]. 

### 6.2. Effects of Contagion Probability and Safety–Productivity Tradeoff

The contagion probability and the safety–productivity tradeoff are two critical drivers concerning the social contagion effect of safety violations within a construction crew. According to the theory of planned behavior [81], an individual’s perceived difficulty in performing a particular behavior (e.g., cutting corners) plays an important role in whether they will adopt the behavior or not. The contagion probability (i.e., median contagionPro) can represent the extent to which a worker follows coworker safety violations within a group context. In contrast, the tradeoff between safety and productivity (i.e., workers compromise safety to achieve effective production) serves as a major system factor that facilitates the formation of safety violation norms [15,19,39,82]. The degree of tradeoff is captured by the potential production increase (i.e., productionIncr) in this research, which reflects the unsuitability between safety and productivity onsite. According to the results of FED analyses, this research found that the contagion probability has significant effects on routine safety violations, while the effects on situational safety violations are relatively lower or even insignificant. This is in line with Liang et al. [18] empirical study, which revealed that routine safety violations are much more contagious than situational safety violations. This is because routine safety violations are more common onsite and they are often committed by experienced workers [83]. By contrast, the safety-productivity tradeoff tends to have a higher effect on situational safety violations than the contagion probability. Different from routine safety violations which are associated with individuals’ cognitive process (e.g., workers commit such violations by completing tasks using the least possible effort) [84], situational safety violations are provoked by the organizational factors such as situational constraints [18,85]. The poor suitability between safety and productivity, reflecting organizational failures of establishing supportive environment with regard to the site, tools or equipment, should have a greater effect on situational safety violations [84]. 

### 6.3. Effects of Different Work Environments

This research examined the effectiveness of four aforementioned managerial interventions on the social contagion effect of safety violations for three different work environment hazard levels. As shown in Section 5, the effects of four managerial interventions on routine safety violations and situational safety violations are much stronger when the hazard level is modest than when the hazard level is low or high (shown in Figure 8b,c, Figure 9b,c and Figure 10b,c). This is because social interactions play a significant role in workers’ decision-making process for the modest hazard situation. Compared with the high or low hazard levels, the modest hazard situation represents a much more ambiguous or uncertain work environment, where individuals are more likely to seek social cues from their coworkers about what behaviors are acceptable within a construction crew. As such, for the modest hazard situation, the contagion probability (i.e., decreasing the median contagionPro) and safety–productivity tradeoff (i.e., decreasing the productionIncr) should be given priority for controlling the social contagion effect. Lastly, considering the significant effects of proactive management strategy, setting safety goals, and their interactive effects (shown in Figure 8b,c), a high intensity of proactive management strategies (i.e., a low proacMan value), and a high safety goal (i.e., a low safeGoal value) are also recommended for the modest hazard environment.

By contrast, it seems that only the work environment plays a critical role for low or high hazard levels. For the low hazard situation, the hazards that workers are exposed to are more likely to be lower than their acceptable level, which leads to workers breaking more safety rules regardless of coworkers’ safety violations. This may be the reason why the effects of the contagion probability and the safety—productivity tradeoff can be so minimal (shown in Figure 10b,c) compared to the effects at modest hazard levels. Moreover, most potential hazards should also be lower than the management tolerable level, which makes management reluctant to provide feedback to safety violations or to take actions to eliminate situational constraints. From this standpoint, highly intensive management strategies (i.e., a low value of proacMan) should be adopted before the occurrence of accidents to ensure a lower tolerable hazard level. 

For the high hazard situation, conversely, the hazards that workers are exposed to are more likely to be higher than their acceptable levels, which will make workers less prone to commit safety violations. As such, the effects of contagion probability and the safety–productivity tradeoff also become limited (Figure 9b,c). Moreover, at a high hazard situation, most potential hazards to which the workers are exposed are so dangerous that they tend to be intolerable to the management. This could explain why implementing a highly intensity of proactive management strategies cannot exert a larger effect on safety violations. In this regard, to further control safety violations, highly intensive proactive management strategies should be supplemented by other interventions. Considering the significant interactive effects between proactive management strategies and safety goals, choosing a high safety goal is also recommended (i.e., a low value of safeGoal) to make the management system much more sensitive to poor safety performance. 

### 6.4. Theoretical Contributions

This research has several theoretical contributions. First, although coworkers serve as a critical source of social influence within a social group context, research on how coworkers’ safety violations influence individuals is still rare [86]. In this regard, this research can extend the current knowledge of safety-related behaviors by modeling the dynamic social contagion effect of safety violations within a construction crew. Second, this research sheds light on how to improve system adaptions to the complexities and uncertainties through dealing with social contagion effect of safety violations onsite. Thus, this research is, to some extent, consistent with the principle of resilience engineering which is a widely used approach to ensure safety performance continuity in changing and uncertain settings [87]. Third, the proposed hybrid SD-ABM simulation approach integrates both the system level dynamics and the individuals’ cognitive process and social interactions. This hybrid model can give a much more accurate representation of the actual system compared to the traditional single methods (i.e., either SD or ABM) that have been often used in construction safety simulations. Fourth, this research employed the FED method to identify the most impactful interventions on safety violations for three different situations (i.e., low-, modest-, and high-hazard situation). The FED can be a much more effective tool than the traditional experimental methods (e.g., Monte Carlo analysis [7]) when the research involves a number of factors and complicated interactions between these factors [3]. Considering that the FED has not received enough attention in current safety-related simulations [3], this research can guide future applications of FED in this research field.

### 6.5. Practical Implications

The findings from this research also have several practical implications. First, the results showed that proactive management strategies can exert much stronger effects on safety violations than reactive strategies (i.e., setting safety goals). Therefore, in addition to setting a high safety goal, organizations should also consider interventions, including having a low tolerable hazard level, providing frequent safety feedback, and inspecting safety widely. Second, management should take different actions to control routine and situational safety violations. For routine safety violations, the tendency to which individuals are influenced by coworkers’ safety violations should be decreased. To reduce contagion probability, workers should be clearly informed that safety rests on everyone including themselves, as they are the ones doing the tasks [82]. When the responsibility for safety can be internalized as a social norm within the crew, the workers may become less susceptible to the norms of safety violations. For situational safety violations, management should improve the suitability of safety procedures to actual workflows; the appropriateness of safety equipment should also be ensured to avoid situations that calls for a tradeoff between safety and productivity [88]. Such a supportive environment not only can be helpful to reduce the situational constraints onsite, but also further motive workers to be active in promoting workplace safety (e.g., assisting others to obtain safety equipment in a timely), which will eventually reduce situational safety violations. Lastly, more attention should be paid to the effects of different working environments (i.e., modest-, low-, and high-hazard situations). For the modest hazard situation, management should give priority to the effects from social interactions by decreasing individual contagion probability and safety–productivity tradeoff. For the low hazard situation, highly intensive safety strategies should be required before the occurrence of injuries or accidents. For the high hazard situation, highly intensive proactive safety strategies should be supplemented by other interventions (e.g., a high safety goal) to further control safety violations. Therefore, this research provides meaningful insight into how to prevent the social contagion effect of safety violations for different hazard levels. 

### 6.6. Limitations and Future Research Directions

First, as Cooke [89] and Sterman [51] have stated, all models are limited and simplified representations of the real world. The SD-ABM model proposed in this research is no exception, especially considering that the hybrid simulation approach is implemented on a virtual construction site. Although such a virtual site is hypothetical and simplified in many aspects, this limitation does not affect the purpose of this research, which is to understand the social contagion effect of safety violations within a construction crew. Thoughtful validation efforts are also conducted in this research to enhance the model. Such efforts include ensuring the agreement between the baseline model and previous empirical data and conducting a sensitivity analysis. In the future, the discrete event simulation (DES), which can capture the construction processes more precisely, is suggested to enhance the proposed SD-ABM simulation [3]. Second, this research assumes that workers have the same productivity rate when they follow safety rules. The assumption of skill homogeneity is acceptable because of the following reasons: (1) the high interdependence in construction tasks means that workers need to cooperate with each other and have similar productivity; and (2) labor productivity is mainly determined by specific project characteristics (e.g., construction technologies, leadership of management, and incentive programs) [90]. Nevertheless, the productivity difference between workers with different working experience (e.g., older and permanent workers tend to be more productive than younger and temporary workers) can be explored in more detail in future research. Third, this research assumes that workers can always perceive hazards and understand what they are being exposed to. However, their ability to recognize hazards can be limited due to various factors, such as personal characteristics and organizational conditions. Construction workers tend to underestimate the actual hazards onsite [91], causing inappropriate decisions. Therefore, this model can be extended by considering a worker’s ability to recognize hazards [5] and other intervention functions, such as accident learning and safety training [2,92], to facilitate a more comprehensive analysis. Fourth, this research mainly focuses on the effect of behavior-related interventions (i.e., safety supervision) without considering other technological investments such as the application of real-time location systems to automatically monitor workers’ behaviors [93]. Therefore, a holistic model that integrates both safety supervision and the application of technology can be established to further explore the effect of the project hazard level on the relationship between the investment in safety and the safety performance [94]. Lastly, more validation studies are suggested in the future by testing the proposed framework on real construction projects. 

## 7. Conclusions

This research developed a hybrid SD-ABM simulation approach to understand the social contagion effect of safety violations within a construction crew based on established social science theories and previous empirical findings. The model confidence was built by implementing verification and validation techniques. Finally, the FED method was used to explore the effectiveness of different interventions for three different situations (low-, modest-, and high-hazard situations). The findings imply that management should take different actions to control routine and situational safety violations. In addition, different interventions should be implemented for different work environments. Specifically, social interactions play a critical role at the modest hazard level because workers may encounter more ambiguity or uncertainty. Interventions such as decreasing the contagion probability or the safety–productivity tradeoff should be given priority. For the low hazard situation, highly intensive proactive management strategies should be required. In contrast, for the high hazard situation, highly intensive proactive safety strategies should be supplemented by other interventions (e.g., a high safety goal) to further control safety violations. Therefore, the findings from this research allow users to select effective interventions to control safety violations at different hazard levels. This research also contributes to the application of the SD-ABM simulation approach to construction safety management.

## Figures and Tables

**Table 1:** Descriptions and equations for variables involved in the SD model.

Variables	Types	Description/Equation
Number of safety improvement	Stock	The total number of safety improvements received by coworkers in an individual’s construction crew
Number of safety feedback	Stock	The total number of safety feedback received by coworkers in an individual’s construction crew
Number of coworker safety violations	Stock	The total number of coworker safety violations in an individual’s construction crew
Perceived safety-specific social support	Intermediate	Perceived safety-specific social support = Min(1, ((Number of safety improvement + Number of safety feedback)/Number of coworker safety violations) × scaling parameter (=100))
Perceived production pressure	Intermediate	If (the productivity of work crew k >= the average productivity of all work crews) Perceived production pressure = 0; If (the productivity of work crew k < the average productivity of all work crews) Perceived production pressure = Min(1, ((the average productivity of all work crews-the productivity of work crew k)/the average productivity of all work crews) × scaling parameter (=100))
Ambivalence toward safety compliance	Output	Attitude ambivalence = min (1, max (0, (0.68 × perceived production pressure-0.13 × perceived safety specific social support))) [18]
Near-miss	Stock	The total number of near-miss incidents caused by safety violations
Accident	Stock	The total number of accidents caused by safety violations
safeGoal (safety goal)	Input	A predefined value for setting the weekly tolerable number of both near-misses and accidents
Safety performance gap	Intermediate	Safety performance gap = (near miss + 10 × accident-safeGoal)/safeGoal
Safety control pressure	Intermediate	If (safety performance gap >= 1) safety control pressure = 1; If (safety performance gap <= 0) safety control pressure = 0; If (0 < safety performance gap < 1) safety control pressure = safety performance gap
proacMan (proactive management strategies)	Input	The proactive safety management, which is different from the reactive actions triggered by the safeGoal, can control the lowest level of intensity of accident intervention measures (i.e., safety improvement rate, safety feedback rate, tolerable hazard level, and distance) before the occurrence of near-misses and accidents.
Safety improvement rate	Output	Safety improvement rate = Max(1-proacMan, safety control pressure)
Safety feedback rate	Output	Safety feedback rate = Max(1-proacMan, safety control pressure)
Distance	Output	Distance = Max(5 × (1-pracMan), 5 × (safety control pressure))
Tolerable hazard level	Output	Tolerable hazard level = Min (100 × proacMan, 100 × (1-safety control pressure))

**Table 2:** Quantitative agreement between the simulation results and empirical data.

Items	Simulation Results	Empirical Data
Ratio of safety violations	0.32	1/3 [75,76]
Proportion of situational safety violations	0.11	0.13 [15]
Ratio between accidents and near-misses	1:8.18	1:10 [77]
Rate of accidents	3.16	3.2 [78]

**Table 3:** Sensitivity analysis of the baseline model.

Model Output	Base Value	Percentage Change of Model Outputs			
		safeGoal	proacMan	median contagionPro	productionIncr
Ratio of safety violations	3.15	+18.55%	+48.52%	+17.56%	+14.71%
Rate of accidents	3.16	+18.95%	+55.79%	+23.16%	+11.58%
Rate of near-misses	33.56	+19.13%	+57.58%	+10.31%	+9.71%
Rate of productivity	19.35	+0.41%	+0.96%	+0.52%	+1.38%

Note: median contagionPro refers to the median value of the contagion probability of all workers.

**Table 4:** The negative and positive levels for the four-factor experiments.

Factors	Negative Level (−)	Positive Level (+)
safeGoal	0.5	2
proacMan	0.2	0.8
median contagionPro	0.2	0.8
productionIncr	0.08	0.32

Note: median contagionPro refers to the median value of contagion probability of all workers.

**Table 5:** Experimental design matrix.

Design Point	safeGoal	proacMan	median contagionPro	productionIncr
1	-	-	-	-
2	+	-	-	-
3	-	+	-	-
4	+	+	-	-
5	-	-	+	-
6	+	-	+	-
7	-	+	+	-
8	+	+	+	-
9	-	-	-	+
10	+	-	-	+
11	-	+	-	+
12	+	+	-	+
13	-	-	+	+
14	+	-	+	+
15	-	+	+	+
16	+	+	+	+

Note: median contagionPro refers to the median value of contagion probability of all workers.

**Table 6:** Average response variables for the “modest” onsite condition.

Design Point	Rate of Accidents	Ratio of Routine Violations	Ratio of Situational Violations	Rate of Productivity
1	1.000	0.052	0.034	19.038
2	1.200	0.081	0.034	19.047
3	2.330	0.213	0.035	19.125
4	5.400	0.405	0.037	19.205
5	1.467	0.086	0.034	19.028
6	2.233	0.121	0.034	19.038
7	4.633	0.355	0.035	19.180
8	7.700	0.544	0.037	19.262
9	1.233	0.058	0.034	19.174
10	1.100	0.074	0.035	19.217
11	2.700	0.253	0.036	19.545
12	4.300	0.424	0.038	19.844
13	1.600	0.131	0.034	19.262
14	1.900	0.161	0.035	19.331
15	4.567	0.375	0.036	19.733
16	6.200	0.560	0.038	20.077

**Table 7:** Average values of response variables for the “high” hazard onsite condition.

Design Point	Rate of Accidents	Ratio of Routine Violations	Ratio of Situational Violations	Rate of Productivity
1	0.933	0.029	0.033	19.014
2	0.900	0.045	0.034	19.016
3	2.533	0.140	0.036	19.088
4	3.533	0.312	0.037	19.138
5	1.033	0.066	0.033	19.035
6	1.433	0.077	0.034	19.048
7	3.333	0.225	0.035	19.125
8	5.367	0.384	0.037	19.204
9	0.933	0.041	0.034	19.117
10	1.033	0.055	0.035	19.167
11	2.433	0.159	0.036	19.361
12	3.933	0.315	0.038	19.639
13	1.533	0.088	0.034	19.196
14	1.367	0.114	0.035	19.253
15	3.967	0.261	0.035	19.475
16	5.833	0.417	0.038	19.786

**Table 8:** Average response variables for the “low” hazard onsite condition.

Design Point	Rate of Accidents	Ratio of Routine Violations	Ratio of Situational Violations	Rate of Productivity
1	0.900	0.090	0.034	19.043
2	1.567	0.132	0.034	19.052
3	3.400	0.368	0.035	19.190
4	7.000	0.643	0.037	19.343
5	2.000	0.176	0.033	19.070
6	2.533	0.254	0.034	19.109
7	6.733	0.578	0.034	19.281
8	10.267	0.775	0.036	19.367
9	1.100	0.115	0.034	19.237
10	1.400	0.150	0.035	19.341
11	3.967	0.384	0.036	19.770
12	6.600	0.704	0.039	20.360
13	3.833	0.272	0.034	19.544
14	4.433	0.437	0.035	19.778
15	7.033	0.606	0.036	20.121
16	10.933	0.879	0.038	20.657
